# Transoral incisionless fundoplication for patients with gastroesophageal reflux disease after peroral endoscopic myotomy: Prospective cohort

**DOI:** 10.1055/a-2681-2538

**Published:** 2025-09-01

**Authors:** Eduardo Guimarães Hourneaux de Moura, Matheus Ferreira de Carvalho, Victor Lira de Oliveira, Mateus Bond Boghossian, Antonio Afonso Miranda Neto, Eduardo Turiani Hourneaux de Moura, André Orsini Ardengh, Ary Nasi, Kenneth Chang, Mateus Pereira Funari

**Affiliations:** 1 Gastrointestinal Endoscopy Unit, Hospital das Clinicas da Faculdade de Medicina da Universidade de São Paulo, São Paulo, Brazil; 2 37870 Surgery, Faculdade de Ciências Médicas da Santa Casa de São Paulo, São Paulo, Brazil; 3 8788 Medicine, University of California Irvine, Irvine, United States; 4 37884 USP, Universidade de São Paulo Faculdade de Medicina, São Paulo, Brazil

**Keywords:** Endoscopy Upper GI Tract, Reflux disease, Motility / achalasia, POEM

## Abstract

**Background and study aims:**

Achalasia is a condition related to failure of relaxation of the lower esophageal sphincter (LES). Treatment is based on reducing LES pressure. Although treatment is traditionally surgical, poor candidates for this modality may be treated with peroral endoscopic myotomy (POEM). However, POEM is associated with a relatively high incidence of gastroesophageal reflux disease (GERD). For cases refractory to proton pump inhibitors (PPIs), transoral incisionless fundoplication (TIF) is one of the endoscopic therapies proposed.

**Patients and methods:**

This was a pilot single-center prospective cohort study including 10 patients with post-POEM GERD refractory to clinical management who underwent endoscopic treatment with the TIF procedure between February and November 2021. We included patients ≥ 18 years old who developed GERD after POEM.

**Results:**

Technical success was achieved in all 10 cases treated with TIF. In 6- and 12-month follow-up, seven patients (70%) reduced PPI use. Two patients (20%) had no esophagitis initially, increasing to five (55%) at 6 months and four (44%) at 12 months. Symptom evaluation and GERD-HRQL questionnaire showed a significant score reduction at 6 months and a downward trend at 12 months. Mean Eckardt score showed a decreasing trend, although mean dysphagia score showed a slight tendency to increase in 1 year. The procedure was considered safe, with no adverse events.

**Conclusions:**

Use of TIF seems to be a feasible alternative for treating GERD after POEM, improving both clinical and endoscopic parameters and pHmetry in a considerable percentage of cases.

## Introduction

Achalasia is an esophageal motility disorder characterized by impaired relaxation of the lower esophageal sphincter (LES) and ineffective peristalsis 1 . Although most cases are idiopathic, Chagas disease is a significant cause in some developing countries. Treatment focuses on disrupting the LES, particularly in patients with severe symptoms, typically indicated by an Eckardt score ≥ 3 2 .

For years, laparoscopic Heller’s myotomy (LHM) has been the standard surgical treatment for most cases 3 . However, with advancements in therapeutic endoscopy and third-space endoscopic techniques, peroral endoscopic myotomy (POEM) is increasingly emerging as a viable alternative 4 5 . Recent trials and meta-analysis have shown clinical success rates of up to 95%, comparable to those for LHM 6 7 8 . However, a major concern with POEM is development of gastroesophageal reflux disease (GERD), because the myotomy of the LES fibers is performed without an accompanying antireflux procedure 9 . Consequently, studies report higher GERD rates and acid exposure time (AET) with POEM compared with LHM (3%-60% vs. 2%-26% and up to 38% vs. 17%, respectively) 10 11 12 .

The literature reports variable success rates for GERD symptom control with clinical management (proton pump inhibitors [PPI], behavioral, and clinical measures) following POEM. Although long-term outcomes show excellent efficacy (up to 100% success after 5 years), patients often experience symptoms during the first years after POEM 13 . Indications for medical therapy remain unclear, because PPIs may be prescribed either for symptomatic relief or esophagitis healing, particularly given frequent discordance between symptoms and endoscopic findings 14 . Furthermore, risk factors for PPI-refractory GERD after POEM are not well established, although some evidence suggests a potential genetic predisposition 15 .

GERD management following POEM presents clinical challenges, with studies showing inconsistent long-term PPI efficacy. Consequently, refractory cases often require additional intervention. Although surgical fundoplication remains the gold standard 16 , post-POEM patients frequently prove suboptimal surgical candidates or decline operative management.

In this context, several endoscopic therapies have emerged as potential treatments for GERD, most notably transoral incisionless fundoplication (TIF). This technique utilizes a specialized device to create a full-thickness, circumferential plication at the gastroesophageal junction (GEJ) using polypropylene fasteners 17 . As an incisionless procedure, TIF minimizes surgical risks while offering an alternative to long-term pharmacotherapy 18 . Clinical studies report success rates up to 90%, with demonstrated improvements in both typical and atypical symptoms. In appropriately selected patients, TIF provides durable symptom resolution, enhanced quality of life, and reduced PPI dependence 19 . Other endoscopic options include antireflux mucosectomy (ARMS) and antireflux mucosal ablation (ARMA), which employ resection or mucosal ablation techniques at the GEJ to reinforce the antireflux barrier. Unlike TIF, these methods do not directly correct anatomical dysfunction. Current evidence for ARMS and ARMA remains limited, highlighting the need for further prospective studies and comparative effectiveness research 20 21 .

Notably, achalasia patients present unique pathophysiologic characteristics due to their underlying esophageal motility disorder. Given limited evidence regarding GERD management after POEM, this study sought to evaluate the efficacy and safety of TIF using the EsophyX Z device (Endogastric Solutions, Redmond, Washington, United States) in this population, with comprehensive therapeutic assessment at 1-year follow up.

## Patients and methods

### Study design

**Fig. 1:**
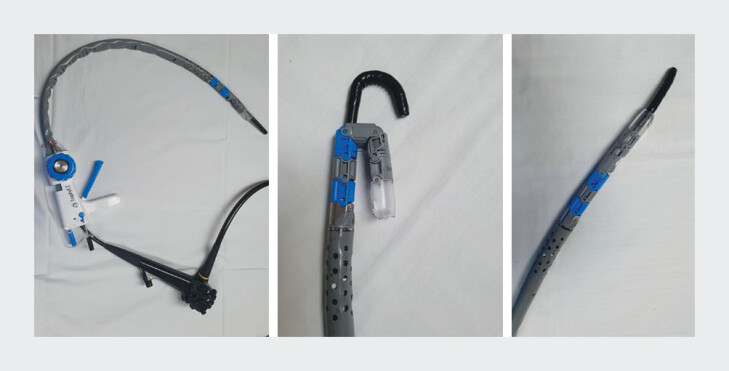
EsophyX Z.

This was a pilot single-center prospective cohort including 10 patients with achalasia and post-POEM GERD refractory to clinical management (drug treatment with omeprazole) who underwent endoscopic treatment with the TIF procedure using the EsophyX Z device between February and November 2021 ( Fig. 1 ). This study was conducted in a tertiary center (Hospital das Clínicas at the University of Sao Paulo Medical School), had institutional review board approval, and was submitted to the Brazilian Clinical Trials Platform prior to enrollment. Because this was a pilot study from inception, the sample size of 10 patients was determined a priori. Inclusion of additional patients was limited by device availability (provided by the equipment supplier) in a resource-constrained setting.

At our institution, all patients with achalasia are diagnosed and classified with esophagogastroduodenoscopy (EGD), high-resolution manometry (HRM), and esophagogram before interventional treatment. In our scenario, some of these patients have Chagas disease, but this does not interfere with the diagnostic criteria used. Every patient who underwent POEM in the last 6 years and presented with reflux disease symptoms despite medical treatment was assessed with EGD, 24-hour pH-monitoring, esophageal manometry, and esophagogram.

### Eligibility criteria

Patients with typical GERD symptoms (heartburn and regurgitation) following POEM (for idiopathic or Chagasic achalasia) were included if they showed objective evidence of pathologic reflux according to 2018 Lyon Consensus criteria: either Los Angeles Grade C/D esophagitis on EGD or AET > 6% on pH monitoring. We also accepted a DeMeester score > 14.7 on pH testing regardless of endoscopic findings, in line with our standard clinical practice 22 . All patients meeting these criteria were eligible for study participation, if they had been using PPIs for at least 8 weeks.

Exclusion criteria were age younger than 18 years, body mass index (BMI) ≥ 30 kg/m ^2^ , hiatal hernia > 2 cm, previous surgical myotomy or fundoplication, persistent dysphagia, weight loss or esophageal bleeding, Barrett’s esophagus, esophageal or gastric varices, esophageal ulcers, delayed gastric emptying, esophageal, gastric or duodenal stenosis or any other upper gastrointestinal tract anatomic alteration that does not allow to perform the procedure, pregnancy, coagulopathy or chronic use of anticoagulants, active neoplasms or any other medical condition that would prevent the patient from completing the follow up. Written informed consent was obtained from all participants.

### Procedure

All procedures were performed by the same endoscopist with significant experience in advanced therapeutic endoscopy, but at the beginning of their TIF learning curve. In the first five cases, the operator was proctored by a senior endoscopist with significant expertise with this technique through real-time videoconferencing. All interventions were conducted under general anesthesia, with CO _2_ insufflation, and patients received antibiotic prophylaxis (intravenous administration of ceftriaxone 1 g and metronidazole 500 mg) before anesthesia induction. Because all patients had achalasia, they remained on 3 days of liquid diet before the procedure and a 12-hour fast.

The procedures began with an EGD, performed with a standard gastroscope (Olympus GIF H-180, Olympus Corporation, Tokyo, Japan) to evaluate the eligibility criteria. Patients were prescribed omeprazole at doses ranging from 40 to 80 mg daily before the TIF procedure.

The procedures were performed with patients in either dorsal or left lateral decubitus position. The EsophyX Z device was loaded with a standard gastroscope and introduced into the stomach. A combined retroflex maneuver of both device and gastroscope was then performed to initiate the procedure. The senior endoscopist operated the device while an assistant manipulated the scope to maintain optimal visualization.

**Fig. 2:**
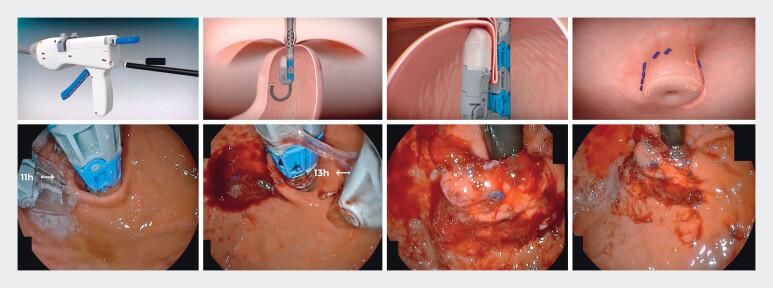
TIF procedure from start to finish. The top figures are the figurative representation, and the bottom figures represent a real patient.

We created a ≥ 2-cm, 270-degree, vertical antireflux flap valve at the GEJ using full-thickness polypropylene fasteners. Valve construction began posteriorly at the 11 o'clock position (3 plications, 6 fasteners) and anteriorly at 1 o'clock (3 plications, 6 fasteners), followed by the greater curvature at 5 and 7 o'clock positions (2 plications and 4 fasteners each). The endoscopist could perform additional firings as needed to optimize valve configuration ( Fig. 2 ).

After the procedure, the patients were kept for clinical observation and were on a liquid diet. They were discharged from the hospital at the end of the same day or in the following morning, depending on pain severity score and liquid diet tolerance.

### Follow-up

After hospital discharge, patients received continuous PPI (pantoprazole 40 mg daily) and oral antibiotics for 7 days (ciprofloxacin 400 mg twice a day and metronidazole 500 mg three times a day) and remained on a soft diet for 14 days with progression to a solid diet after this. Post-procedural follow-up visits were scheduled for all patients after 1, 3, 6, and 12 months, with EGD, application of the GERD-HRQL questionnaire, and Eckardt score. Additionally, 24-hour pH monitoring was performed at 6 and 12 months.

### Outcomes and definitions

The primary outcome was symptomatic improvement, defined as > 50% reduction in GERD-health-related quality of life (HRQL) questionnaire scores 23 . Secondary outcomes included: PPI dose reduction or discontinuation, LA grade of esophagitis on EGD, Eckardt score and its dysphagia component 24 , and pH-monitoring parameters (AET% and DeMeester scores). All the results were manually reviewed. GERD diagnosis followed 2018 Lyon Consensus criteria throughout the study 22 .

One patient became pregnant during follow-up and consequently completed only the 1-year EGD and pH-monitoring assessments. A second patient withdrew after 6 months due to severe reflux symptoms requiring surgical referral.

### Statistical analysis

Categorical variables were described as absolute values of frequency, proportions, and percentages. All statistical analyses were performed using Jamovi 2.6 software (The Jamovi Project, 2025) including the Friedman test for repeated measures with the Durbin Conover post hoc test. Categorical variables were analyzed using the two- proportion test.

## Results

Ten patients with post-POEM GERD underwent a TIF procedure. Baseline characteristics of the study population are summarized in Table 1 . In six patients (60%), the achalasia diagnosis was secondary to Chagas disease and five (50%) had LA grade C or D esophagitis. All procedures (100%) achieved technical success with no adverse events (AEs). Eight patients (80%) were discharged from the hospital at the end of the procedure day, whereas the remaining two were kept for observation and were discharged the morning after the procedure: one for limited dysphagia with spontaneous resolution and another patient for mild abdominal pain, controlled with basic analgesics. Intraprocedural and early post-procedural outcomes are summarized in Table 2 .

**Table:** **Table 1** Population study baseline characteristics.

**Characteristics**	**Results**
Mean age (± SD)	53.8 (13.7)
Gender, n (%)	
Male	4 (40)
Female	6 (60)
Mean BMI (± SD)	26.5 (4)
Etiology, n (%)	
Idiopathic	4 (40%)
Chagas disease	6 (60%)
Mean time from POEM, years (± SD)	3.4 (1.6)
Los Angeles Grade esophagitis, n (%)	
Absent	2 (20)
Grade A	1 (10)
Grade B	2 (20)
Grade C	3 (30)
Grade D	2 (20)
HRM classification, n (%)	
Type I achalasia	8 (80)
Type II achalasia	2 (20)
Mean acid exposure time % (± SD)	21.6 (14.1)
Esophagogram (classification of Rezende), n (%)	
Grade I	3 (30)
Grade II	4 (40)
Grade III	3 (30)
Daily PPI use (%)	10 (100)
BMI, body mass index; HRM, high-resolution manometry; POEM, peroral endoscopic myotomy; PPI, proton pump inhibitor; SD, standard deviation.	

**Table:** **Table 2** Intraprocedural and early post-procedural outcomes.

**Outcomes**	**Results**
Technical success, n (%)	10 (100)
Adverse events, n (%)	0 (0)
Mean procedure time, minutes (± SD)	59 (26)
Mean length of hospitalization, days (± SD)	1.2 (0.4)
SD, standard deviation.	

After the TIF procedure, three patients (30%) persisted with daily PPI use (40 mg omeprazole) on both the 6- and 12-month evaluations. Mean AET showed a downward trend on the 6-month evaluation (21.6% ± 14.1% vs. 11% ± 11.5%) and on the 12-month evaluation (21.6% ± 14.1% vs. 12.7% ± 11.7%). The number of patients with AET > 6% also showed a downward trend both on the 6-month evaluation (70% vs. 55%) and on the 12-month evaluation (70% vs. 44%). DeMeester score dropped on the 6-month follow-up (80 ± 49 vs. 42.6 ± 45.8) and also on the 12-month follow-up (80 ± 49 vs. 49.2 ± 44.5). The number of patients with DeMeester score > 14.7% also showed a downward trend both on the 6-month evaluation (90% vs. 55%) and on the 12-month evaluation (90% vs. 44%). All patients with an abnormal DeMeester score also had altered AET on both the 6-month and 12-month follow-ups, demonstrating that both parameters followed the same trend, despite the confounding factors of the DeMeester score in patients with achalasia. Symptom evaluation and quality of life measured by the GERD-HRQL questionnaire demonstrated a significant reduction in mean score values on the 6-month follow-up (27.8 ± 10 vs. 13.7 ± 11.5), and a drop on the 12-month follow-up (27.8 ± 10 vs. 15.2 ± 11.5). On the 6 and 12-month follow-up, seven patients (70%) reduced their PPI use. Regarding LA grade esophagitis, only two patients (20%) showed no esophagitis on the initial evaluation. On the 6-month evaluation, this number increased to five (55%), and at 12 months it was four (44%). Mean Eckardt score showed a decreasing trend both on the 6-month (3.5 ± 1.43 vs. 2,7 ± 2.15) and 12-month (3.5 ± 1.43 vs. 2.6 ± 1.68) evaluations. It was observed that mean dysphagia score (from Eckardt) showed a slight tendency to increase both on the 6-month (0.8 ± 0.6 vs. 1.5 ± 0.92) and 12-month (0.8 ± 0.6 vs. 1.1 ± 0.62) follow-ups. However, neither the change in dysphagia nor the change in the Eckardt score reached statistical significance. It is important to highlight that the included patients had undergone POEM at least 2 years prior to our study, with confirmed treatment efficacy during this period. In addition, the pre-TIF Eckardt score was predominantly attributed to the reflux component. Table 3 compares main outcomes 6 months and 12 months after the TIF procedure with pre-procedure baseline characteristics and Table 4 highlights the percentage of patients that presented with improvement of these outcomes at 12 months.

**Table:** **Table 3** Comparison of outcomes between pre-TIF and 6 and 12 months post-TIF procedure.

**Outcomes**	**Pre-procedural**	**6-month follow up**	**12-month follow up**
Daily PPI use, n (%)	10 (100)	3 (30) (P = 0.754)	3 (30)
Los Angeles Grade esophagitis, n (%)			
Absent, n (%)	2 (20)	5 (55)	4 (44)
A	1 (10)	0 (0)	0 (0)
B	2 (20)	1 (11)	2 (22)
C	3 (30)	2 (22)	3 (33)
D	2 (20)	1 (11)	0 (0)
Mean AET% (± SD)	21.6 (14.1)	11 (11.5)	12.7 (11.7)
Patients with AET > 6%, n (%)	7 (70)	5 (55)	4 (44)
Mean DeMeester Score (± SD)	80 (49)	42.6 (45.8)	49.2 (44.5)
Patients with DeMeester Score > 14.7, n (%)	9 (90)	5 (55)	4 (44)
Mean GERD-HRQL Score (± SD)	27.8 (10)	13.7 (11.5)	15.2 (11.1)
Mean Eckardt score	3.5 (1.43)	2.7 (2.15)	2.6 (1.68)
Mean dysphagia score (from Eckardt)	0.8 (0.6)	1.5 (0.92)	1.1 (0.62)
AET, acid exposure time; GERD-HRQL, gastroesophageal reflux disease health-related quality of life; NA, not applicable (due to the low number of cases under review); PPI, proton pump inhibitor; SD, standard deviation; TIF, transoral incisionless fundoplication.			

**Table:** **Table 4** Percentage of outcomes improvement post-TIF procedure after 12-month follow-up.

**Outcomes**	**Results**
PPI use reduction, n (%)	7 (70)
Downgrade Los Angeles Grade esophagitis, n (%)	6 (67)
AET% reduction, n (%)	6 (67)
GERD-HRQL reduction, n (%)	7 (70)
AET, acid exposure time; GERD-HRQL, gastroesophageal reflux disease health-related quality of life; PPI, proton pump inhibitor; TIF, transoral incisionless fundoplication.	

## Discussion

This is a study that adds information about a treatment that is not yet fully well established. When our protocol was designed, some case reports and retrospective studies were available in the literature 25 26 . Recently, another prospective cohort was published on the same topic, however, 88% of the cases had TIF combined with laparoscopic hiatal hernia repair 27 . The efficacy and safety of the TIF procedure in the general population with GERD is well established, as demonstrated by previous randomized controlled trials and meta-analysis 28 . Recently, a prospective study was published evaluating the efficacy of TIF in post-POEM patients with GERD as a first-line treatment. In this study, it was observed that TIF was safe and led to comparable improvements in GERD-HRQL scores when compared with long-term PPI therapy 29 . Also, some other retrospective studies were conducted in the subgroup of patients with post-POEM GERD with similar positive outcomes 23 24 . In our prospective study, we had 100% technical success with no AEs, reinforcing the feasibility and safety of this technique.

It is essential to note that many patients with achalasia treated with POEM, especially those with Chagas disease, are poor candidates for surgery (LHM), or have previously refused surgical treatment. In this context, TIF is a valuable alternative that fills an important gap between clinical and surgical treatment, offering an interventional, but less invasive therapy with a good safety profile.

Regarding GERD control, there was a drop in PPI dosage and GERD-HRQL scores in 70% of the patients (n = 7), and a trend of improvement in LA grade esophagitis and AET% values in 67% (n = 6). Although the results in the available literature appear to be slightly better, there is a great variance in outcomes analyzed post procedure 25 26 30 .

Defining clinical success in GERD treatment remains challenging due to inconsistent parameters in the literature. Available studies typically assess three key domains: clinical features (including PPI reduction/discontinuation and symptom questionnaires), endoscopic findings (such as esophagitis healing), and pH-monitoring results (AET%, reflux duration, and DeMeester scores). Although these assessment methods vary, most post-TIF studies report better outcomes for subjective measures such as symptom improvement and PPI reduction 31 compared with objective pH-study parameters 18 . In this prospective cohort, most patients (70%) had a reduction in PPI usage and GERD- HRQL scores (pre-TIF mean 27.8 ± 10 vs. post-TIF mean 15.2 ± 11.1), but only 40% had a reduction > 50% in these values. Although most patients (67%) had a downgrade of esophagitis, only 40% developed esophagitis healing. Furthermore, despite the AET% values reduction in 67% of the patients, the mean post-TIF value (12.7 ± 11.7) was still high, and only four patients (44%) had normal values of AET% (< 6%) at 1-year follow-up. No significant difference in the Eckardt score was observed before and after the treatment.

For patients with minimal GERD symptoms and LA grade A or milder esophagitis, the clinical outcome represents meaningful therapeutic success - regardless of PPI use status. This interpretation holds particular importance, given their underlying physiological challenges, including prolonged acid exposure and impaired esophageal clearance.

This study has some limitations including that all procedures were performed by a single faculty member at the beginning of his learning curve in a single center. This may have contributed to suboptimal outcomes, even though it also made our results more widely applicable. Esophageal hypersensitivity, gastroparesis, technical factors during procedure execution, and poor post-procedural compliance may also contribute to treatment failure. An additional limitation stems from our small sample size, inherent to this pilot study's rigorous inclusion criteria. In addition, most of the patients (60%) had Chagas disease as etiology of achalasia, which is different from the epidemiology in the literature of non-emerging countries. Nevertheless, this is unlikely to have had a negative impact on the outcomes because the disease’s pathophysiology and progression is similar to that for primary achalasia 32 . This study used the DeMeester score for pre- and post-TIF evaluation of patients. Although it is not validated for patients with achalasia in the Lyon Consensus, it is a score widely used in our center and should be interpreted with caution in this context. Another limitation lies in the difficulty in assessing symptomatic improvement, because achalasia symptoms such as regurgitation or retrosternal pain can be confounding factors and lead to worse score values in questionnaires such as GERD-HRQL when compared with the general population. pH monitoring requires particularly careful interpretation in achalasia patients undergoing TIF, both pre- and post-procedure. The impaired esophageal clearance in these patients creates two distinct mechanisms of esophageal acidification: prolonged retention of ingested acidic materials and fermentation of stagnant food contents. This dual pathophysiology necessitates cautious analysis of pH-study results to avoid misinterpreting these phenomena as true gastroesophageal reflux events. These two factors significantly compromise analysis of actual occurrence of gastroesophageal acid reflux. Prolonged monitoring of reflux by impedance-pH monitoring also does not clarify this issue. With stagnant material in the esophagus, the esophageal baseline impedance is greatly reduced, making reflux analysis difficult. Therefore, what we actually assess in these methods is the percentage of intraesophageal acidification which, as we have seen, does not adequately reflect reflux episodes 33 .

Based on our findings and available data in the literature, TIF represents a feasible therapeutic option for managing GERD following POEM. However, optimal patient selection and objective pH-monitoring testing confirming pathologic reflux are mandatory before an interventional decision, in order to achieve best outcomes. Although patients with refractory post-POEM GERD who required long-term continuous PPI use should be considered for invasive therapies, those with intense symptoms or LA grade C or D despite high-dose PPIs also have worse outcomes and tend to be non-responders 34 . Furthermore, TIF should not be performed in patients with > 2 cm hiatal hernias or gastroesophageal flap valve Hill grade III or IV as an isolated therapy. Nevertheless, recent studies have demonstrated good outcomes with combined therapy (concomitant surgical hiatal hernia repair and TIF) in this subgroup of patients 35 . Despite positive initial studies, randomized trials will provide important additional data, and help to provide optimal patient selection.

## Conclusions

In summary, this prospective, single-center, cohort study found that TIF represents a feasible therapeutic option for managing GERD following POEM, although conclusive evidence of its efficacy remains limited. It effects a decrease in PPI dosage in most patients as well as a reduction in mean values of GERD-HRQL, AET%, Eckardt score, and grade of esophagitis. However, some patients may notice a slight worsening of dysphagia and only a few patients can expect normalization of AET or complete healing of esophagitis.
